# Staging CT and Diagnostic Laparoscopy With Cytology Prior to the Treatment of Pancreatic Adenocarcinoma: A Case Series

**DOI:** 10.7759/cureus.31883

**Published:** 2022-11-25

**Authors:** Neal S Panse, Vishnu Prasath, Simran Arjani, Patrick L Quinn, Ronak Trivedi, Ravi J Chokshi

**Affiliations:** 1 Surgical Oncology, Rutgers University New Jersey Medical School, Newark, USA; 2 Internal Medicine, Montefiore Medical Center, Wakefield Campus, Bronx, USA; 3 Surgery, The Ohio State University Wexner Medical Center, Columbus, USA

**Keywords:** r0 resection, peritoneal cytology, diagnostic laparoscopy, tumor staging, pancreatic adenocarcinoma treatment

## Abstract

Introduction: Initial staging of pancreatic ductal adenocarcinoma (PDAC) is performed with computed tomography (CT). Laparoscopy with peritoneal cytology at staging can uncover occult disease undetected by CT. This case series assessed clinical course following staging laparoscopy with cytology in patients with PDAC.

Methods: This single-center study examined patients with non-metastatic PDAC diagnosed from 2017 to 2020. Patients underwent CT and subsequent laparoscopy with cytology prior to treatment. Demographics, clinicopathologic status, treatment course, and survival were compared.

Results: Eight patients were identified. All had negative laparoscopies. Five cytologies were negative, two were atypical, and one was positive. Two patients with negative cytology received neoadjuvant chemotherapy and underwent resection, with an average follow-up time of 32.9 months since diagnosis. Of the three remaining patients with negative cytology, none underwent resection. One received delayed chemotherapy, while the others could not due to medical contraindications. The average survival was 3.5 months (n=2). Of two patients with atypical cytology, neither underwent resection. One could not receive chemotherapy due to medical contraindication, while the other was lost to follow-up shortly after diagnosis. The average survival was 1.3 months (n=1). The patient with positive cytology received definitive chemotherapy without resection and survived for 21.6 months.

Conclusions: The patient with positive cytology may have been spared non-therapeutic surgery. Remaining unresected patients showed poor survival, though the lack of immediate chemotherapy may contribute to this finding. Further research is needed to determine optimal candidates for invasive staging and implications of atypical cytology.

## Introduction

Pancreatic cancer has a five-year survival rate of approximately 10%, and incidence has been trending upward for the past 20 years [1,2]. Margin-negative (R0) resection remains the only potentially curative therapy. Per the 2021 National Comprehensive Cancer Network (NCCN) guidelines, systemic therapy and surgery are recommended for patients with localized disease, whereas definitive chemotherapy is recommended for patients with metastases [3]. Initial staging is commonly performed with computed tomography (CT) scan. However, despite recent advances in CT capabilities, it may be falsely negative in patients with metastatic lesions smaller than approximately 1 mm or peritoneal micro-metastases [4]. The use of CT alone for staging may therefore lead to overtreatment with non-therapeutic surgical resection in some patients who should not be candidates for curative therapy [5,6].

To improve the accuracy of staging, diagnostic laparoscopy with peritoneal cytology may be performed before the initiation of systemic therapy and surgical resection. This method of comprehensive staging prior to neoadjuvant therapy is a relatively new treatment algorithm and was recently adopted by our program. Laparoscopy is used to identify gross metastatic lesions; meanwhile, cytologic examination can identify peritoneal micro-metastases imperceptible to both CT and laparoscopy at minimal additional morbidity [6,7]. The presence of gross or micro-metastatic lesions classifies the disease as stage 4, limiting treatment options to systemic and palliative therapies and portending poor survival [8,9]. Evidence from published literature suggests that for disease classified as resectable by NCCN criteria, positivity rates of laparoscopy alone may be as high as 27% and may be even higher in more advanced tumors [6,10,11]. Thus, the addition of laparoscopy with cytology may more accurately stage patients who would otherwise be subjected to unnecessary therapies.

Despite theoretical benefits of more accurate staging, there is no clear consensus on which patients should undergo laparoscopy with cytology and whether treatment based on laparoscopy with cytology improves survival [12]. Some argue against universal invasive staging due to risks associated with an additional laparoscopy and due to decreasing yield as CT technology improves [13]. There are few studies examining outcomes of laparoscopy with cytology prior to the initiation of neoadjuvant chemotherapy. We sought to describe the treatment course of our patients with biopsy-proven pancreatic adenocarcinoma who underwent staging laparoscopy with cytology in addition to the standard staging CT and to contextualize our results within the limited literature.

## Materials and methods

This is a single-institution case series of patients treated at University Hospital in Newark, New Jersey, for biopsy-confirmed pancreatic adenocarcinoma from 2017 to 2020. Only biopsy-proven cases were considered to avoid including patients with benign pancreatic masses or neuroendocrine tumors. Records were prospectively maintained and were reviewed for all documentation until November 2022. Patients first received a diagnostic CT, followed by endoscopic ultrasound-guided fine-needle aspiration (EUS-FNA) biopsy to confirm malignancy. Per NCCN criteria, resectable tumors are contained within the pancreas without local extension, borderline resectable tumors contact local structures but can be surgically resected, and locally advanced tumors abut local structures and may not be amenable to resection without demonstrable response to chemotherapy first [3]. Appropriate surgical candidates with resectable, borderline resectable, or locally advanced disease were offered diagnostic laparoscopy with cytology. All invasive staging procedures were performed prior to the initiation of systemic therapy. Individuals without metastases after complete staging and without medical contraindications were initiated on neoadjuvant chemotherapy. The preferred regimen was leucovorin, 5-fluorouracil, irinotecan, and oxaliplatin (FOLFIRINOX). After the completion of neoadjuvant therapy, surgical candidates underwent resection.

All laparoscopies and resections were performed by a single surgeon. Patients who deferred laparoscopy with cytology, underwent exploratory laparotomy for initial staging, or were treated with a surgery-first approach were excluded. Demographics, clinicopathologic status, treatment course, and survival were obtained and analyzed for included patients. Patients with borderline resectable and locally advanced disease were classified as locoregional in this analysis. The results of laparoscopy with cytology were compared based on NCCN resectability status as per initial CT. Outcomes including the ability to tolerate chemotherapy, resection status, and overall survival were compared stratified by results of invasive staging. Given that this study involved a review on patient records, Institutional Review Board (IRB) approval was obtained prior to data extraction (approval number Pro2011001092). Authors NSP, VP, RT, and RJC were included on the IRB and take responsibility for the integrity of the data. All other authors received de-identified versions of the included data.

## Results

Eleven patients with pancreatic adenocarcinoma were identified, eight of whom met the study criteria. The average age at diagnosis among included patients was 65.8 years old (Table 1). Four patients (50%) were male, and six (75%) identified as non-White. Common comorbidities included hypertension (75%) and diabetes mellitus (75%). Seven patients had tumors involving the pancreatic head, while two patients had diseases involving the body or tail. One of these patients had tumor involvement of the head, body, and tail. One patient had a resectable disease by initial CT (12.5%), while seven had locoregional disease (87.5%).

**Table 1:** Patient demographics Demographic information was obtained from chart review and was either self-identified or obtained by healthcare providers. Age is presented in years at the time of tissue diagnosis. Sex refers to biologic sex. Race is classified as White or non-White. Comorbidities and surgical history were assessed at the time of tissue diagnosis. Smoking history was classified as never, former, or current smoker. Patients were considered to have a history of alcohol use if they had used any amount of alcohol at or prior to tissue diagnosis HTN: hypertension; DM: diabetes mellitus; HLD: hyperlipidemia

Patient ID	Age at Diagnosis (Years)	Sex	Race	Comorbidities	Surgical History	Smoking Status	History of Alcohol Use
A	66	Female	Non-White	HTN and DM	Cesarean section (unspecified year), unspecified left knee surgery (2011), and unspecified right hip surgery (2013)	Former	Yes
B	64	Female	Non-White	HTN and DM	Thyroidectomy (unspecified year) and tonsillectomy (unspecified year)	Never	Yes
C	63	Male	Non-White	HTN and DM	None	Never	No
D	56	Male	Non-White	None	None	Former	Yes
E	64	Female	Non-White	HTN, DM, and HLD	None	Never	No
F	77	Male	White	HTN and DM	None	Former	Yes
G	62	Female	Non-White	DM	None	Never	No
H	74	Male	White	HTN and HLD	Tonsillectomy (1948) and bilateral inguinal hernia repair (1990)	Former	No

Baseline tumor characteristics are presented in Table 2.

**Table 2:** Clinicopathologic characteristics at diagnosis Clinicopathologic characteristics of each patient were obtained from a retrospective chart review. Patient information was de-identified and is organized by National Comprehensive Cancer Network (NCCN) resectability status. Presenting symptoms listed were included in notes from the first diagnostic visit, and findings not being listed do not rule out their presence. Tumor location, maximum diameter, and the presence of enlarged lymph nodes were assessed on the first diagnostic CT. Resectability is based on NCCN criteria. Baseline CA 19-9 level indicates the first measured CA 19-9 during diagnostic workup ^a^Reference range 0-35 units/mL ^b^Initial CT documents that pancreatic size was 14 cm × 4.5 cm with a mass in the head, body, and tail but does not give dimensions of the mass CT: computed tomography; LFTs: liver function tests; CA: cancer antigen

Patient ID	Presenting Symptom(s)/Finding(s)	Tumor Location	Maximum Tumor Diameter (cm)	Initial CT Resectability	Enlarged Lymph Nodes on Initial CT	Baseline CA 19-9 (units/mL)^a^
A	Abdominal pain, pruritis, dark urine, and weight loss	Head	2.8	Resectable	No	79
B	Epigastric pain and elevated LFTs	Head	3.8	Locoregional	Yes	<2.0
C	Abdominal pain and jaundice	Head	4.0	Locoregional	Yes	36
D	Epigastric pain	Head, body, and tail	Not reported^b^	Locoregional	Yes	1,048
E	Epigastric pain	Body/tail	3.1	Locoregional	No	344
F	Abdominal pain, jaundice, and emesis	Head	2.7	Locoregional	Yes	1,655
G	Abdominal pain, jaundice, and weight loss	Head	6.9	Locoregional	Yes	2,329
H	Abdominal pain and emesis	Head	3.5	Locoregional	No	1,157

All laparoscopies were negative for gross metastasis (Table 3). The patient with resectable disease (patient A) and four patients with locoregional disease (patients B-E) had negative cytologies as well. Of the remaining three patients with locoregional disease, two cytologies showed atypical cells (patients F and G), and one was positive for micro-metastases (patient H). Among five total patients with a negative laparoscopy and negative cytology (patients A-E), two underwent surgical resection (patients A and B). No surgical resections were aborted mid-procedure due to disease spread. None of the three patients with atypical or positive cytology underwent resection.

**Table 3:** Treatment outcomes and survival data Data was obtained via retrospective chart review. Resectability status is based on the initial CT image and National Comprehensive Cancer Network guidelines. Laparoscopy was negative if there was no evidence of gross metastasis. Peritoneal cytology samples were taken at the time of laparoscopy and assessed histologically. Neoadjuvant chemotherapy regimen with a number of cycles or contraindication to neoadjuvant therapy is listed. Patients who underwent resection were identified, and margin status is presented. Survival is presented in months if complete data is available; otherwise, time to the last follow-up is reported ^a^Reported survival is time to last follow-up ^b^Regimen described here is definitive chemotherapy without plan for future resection CT: computed tomography; R0: margin-negative; FOLFIRINOX: leucovorin, 5-fluorouracil, irinotecan, and oxaliplatin

Patient ID	Initial CT Resectability	Laparoscopy Results	Cytology Results	Neoadjuvant Chemotherapy Regimen	Resection (Yes/No)	Survival (Months)
A	Resectable	Negative	Negative	FOLFIRINOX (eight cycles)	Yes (R0)	28.3^a^
B	Locoregional	Negative	Negative	FOLFIRINOX (12 cycles)	Yes (R0)	37.4^a^
C	Locoregional	Negative	Negative	None (due to concomitant cholangitis)	No	0.9
D	Locoregional	Negative	Negative	FOLFIRINOX (eight cycles)	No	6.1
E	Locoregional	Negative	Negative	None (due to concomitant appendiceal abscess)	No	2.3^a^
F	Locoregional	Negative	Atypical	None (due to liver enzyme elevation)	No	1.3
G	Locoregional	Negative	Atypical	Unknown	No	0.4^a^
H	Locoregional	Negative	Positive	Gemcitabine with paclitaxel (three cycles) and gemcitabine alone (one cycle)^b^	No	21.6

Patients A and B received neoadjuvant FOLFIRINOX prior to R0 resection and remain alive, with follow-up time of 28.3 and 37.4 months since diagnosis and disease-free survival of 20.7 and 28.8 months, respectively (Table 4). Three patients (patients C-E) had negative laparoscopy and cytology but did not undergo resection. Patient C was unable to receive chemotherapy due to complications secondary to cholangitis and survived for 0.9 months. Patient D received FOLFIRINOX and survived for 6.1 months. This patient deferred biopsy for two months after initial CT, which likely delayed the initiation of chemotherapy. Patient E was unable to receive chemotherapy due to complications related to an appendiceal abscess. This patient was discharged to home hospice care after developing malignant ascites, and no further follow-up information was available. Among the two patients with negative laparoscopy and atypical cytology, Patient F was unfit to receive chemotherapy due to elevated liver enzymes despite adequate performance status and survived for 1.3 months. Patient G was lost to follow-up shortly after diagnostic laparoscopy with cytology. The patient with negative laparoscopy and positive cytology (patient H) initially received gemcitabine with paclitaxel and later switched to gemcitabine alone due to intolerable fatigue. This patient survived for 21.6 months.

**Table 4:** Survival time by invasive staging results and resection status, excluding patients lost to follow-up Survival data was obtained via retrospective chart review and was stratified by results of laparoscopy with cytology and by resection status. Survival is reported only in patients with complete survival data. Overall survival represents time from tissue diagnosis to death or time to last follow-up in patients who remain alive. Disease-free survival represents time from resection to last follow-up without disease recurrence ^a^Reported survival is time to last follow-up

	Laparoscopy and Cytology Negative and No Resection (n=2)	Laparoscopy Negative and Atypical Cytology (n=1)	Laparoscopy Negative and Cytology Positive (n=1)	Laparoscopy and Cytology Negative and Resection Performed (n=2)
Overall survival, months (range)	3.5 (0.9-6.1)	1.3	21.6	32.9 (28.3-37.4)^a^
Disease-free survival, months (range)	--	--	--	24.8 (20.7-28.8)

## Discussion

The purpose of our study was to explore the possible benefits of adding diagnostic laparoscopy with peritoneal cytology to staging CT prior to the initiation of neoadjuvant therapy in patients with both resectable and locoregional pancreatic adenocarcinoma. In this single-center study, eight patients with non-metastatic pancreatic cancer who underwent laparoscopy with cytology were identified. Laparoscopy, independent of cytology, did not provide additional staging information. Atypical peritoneal cytology was associated with short survival time and may be indicative of poor prognosis. One patient with positive cytology received definitive chemotherapy only and showed excellent response. Regardless of tumor stage or the use of invasive staging techniques, patients who did not receive systemic therapy and were not resected had the worst survival.

This case series was pursued because there are very few published studies examining clinical outcomes in patients with pancreatic cancer who undergo staging laparoscopy with cytology after initial CT and prior to systemic therapy. One such retrospective cohort study, which examined 75 patients with CT-diagnosed borderline resectable disease, found occult metastases in 25% of patients who underwent laparoscopy with cytology [11]. The R0 resection rate in those subsequently receiving neoadjuvant therapy was 37% [11]. No other similar cohort studies or randomized controlled trials were identified in the literature. Multiple other studies have demonstrated improved detection of metastatic disease via laparoscopy with cytology, but these studies either do not report postsurgical outcomes or involve upfront surgery, a treatment method that is falling out of favor [14-16]. While our study has a small sample size, our patient-specific data may provide insights into an understudied treatment algorithm. Our results highlight that laparoscopy with cytology may be of use based on a patient’s probability of having micro-metastases, that the significance of atypical cytology requires further study, and that while invasive staging may help determine whether resection should be performed, the initiation of systemic therapy may have a greater impact on survival.

There is much debate regarding the use of laparoscopy and cytology as staging tools and which patients should receive them. In our study, laparoscopy results were negative in all patients, but cytology revealed atypical or positive results in three patients (patients F-H). The addition of cytologic examination to laparoscopy therefore improved the sensitivity of invasive staging. Despite advancements in CT imaging, peritoneal cytology is able to identify micro-metastases not detected by other staging techniques [17]. The percentages of laparoscopy-negative, cytology-positive patients are as high as 11.5%-21.4% in some studies [18,19]. For patients with localized tumors, high-potency neoadjuvant therapy may downstage disease and increase R0 resection rates [20]. However, without invasive staging, patients with positive cytology may undergo upfront surgical resection, which would delay systemic therapy and could negatively affect survival [7,21]. In our study, patient H was identified as cytology-positive in the absence of gross metastases and was treated with systemic therapy alone. The use of invasive staging helped guide optimal therapy and avoided a potentially futile resection that could have also reduced the patient’s quality of life [21].

The 2021 NCCN guidelines suggest performing staging laparoscopy in high-risk cases [3]. However, as the sensitivity of CT imaging continues to improve and as patients with disease not classified as resectable increasingly receive high-potency neoadjuvant chemotherapy before pre-surgical restaging, the role of invasive staging remains unclear [3,20,22]. One 2016 meta-analysis suggests that patients with initial cancer antigen (CA) 19-9 >150 units/mL or tumor size >3 cm would benefit from staging laparoscopy [12]. Other proposed high-risk characteristics include locally advanced disease and, to a lesser extent, borderline resectable disease or tumor originating from the pancreatic body or tail [23]. In our study, there were five patients with CA 19-9 >150 units/mL (patients D-H). Additionally, patients B and C had locally advanced disease with tumors larger than 3 cm and therefore were at high risk for occult metastases as well. Among these seven high-risk individuals, one was cytology-positive, and two had atypical cytology, suggesting that the yield of invasive staging could be as high as 43%. Thus, the positivity rate of staging laparoscopy with cytology may be significant enough with certain high-risk features to warrant use in these cases.

There is limited literature regarding outcomes in patients with atypical peritoneal cytology obtained after biopsy-proven diagnosis of pancreatic adenocarcinoma. Among the two patients with atypical cytology in this study, patient F had a short survival of 1.3 months. Patient G was lost to follow-up shortly after diagnostic workup, though this patient had the largest documented tumor size and highest baseline CA 19-9 of any patient in our study. In one published study, cytology samples were obtained from either EUS-FNA or biliary brushing in patients with suspicious pancreaticobiliary masses, and these samples were assessed histologically [24]. Subsequent mutational analysis performed on atypical samples showed evidence of underlying aggressive malignancy in 89% of cases [24]. Another study found that atypical cytology samples taken during endoscopic retrograde cholangiopancreatography for the evaluation of suspicious biliary strictures had a false-negative rate of 80% [25]. These studies and our results concur that atypical peritoneal cytology may represent a falsely indeterminate result in the setting of micro-metastases.

Our study also suggests that regardless of initial staging, the initiation of chemotherapy and response to systemic therapy may have significant prognostic implications. The average survival of patients who did not receive chemotherapy (patients C and F) was 1.1 months, while those receiving chemotherapy without resection (patients D and H) had survival of 6.1 and 21.6 months, respectively. Notably, patient D likely received delayed chemotherapy, which has been associated with poor outcomes and may have reduced survival in this patient [26]. Evidence from the literature suggests that adequate chemotherapy, independent of resection status, can increase progression-free survival, which strongly correlates with increased overall survival [27]. There also appears to be a greater survival benefit with higher potency regimens than lower potency regimens, even in patients with locally advanced and metastatic tumors [20,28,29]. Since the use of high-potency chemotherapy is dependent on optimal performance status, considering a patient’s functional capacity could help guide the use of invasive staging. In patients who could tolerate high-potency chemotherapy, diagnostic laparoscopy with cytology may not alter disease management. For patients with poor performance status, the positivity of invasive staging could allow providers to maximize quality of life through the avoidance of high-toxicity therapies or attempted curative resection [30]. Until chemotherapy regimens capable of more accurately targeting pancreatic adenocarcinoma while minimizing side effects are available, invasive staging may still provide clinical value.

The primary limitation of this study was its small sample size, which increases the risk of sampling bias and limits statistical power. This also limited our ability to perform statistical analyses or draw definitive conclusions from our results. A larger cohort study or randomized controlled trial would be better suited to assess hypotheses generated by our study. The authors chose to present data individually for each patient rather than in mean values to preserve the integrity of the work; presenting data as a mean may be misleading with a small sample size. The retrospective nature of this study increases the risk of information bias due to the possibility of poor documentation or missing information. This study also lacked complete follow-up and survival data for two patients (patients E and G), which may have altered results.

## Conclusions

Our study suggests that the addition of laparoscopy with cytology to pancreatic adenocarcinoma staging in high-risk patients may spare non-therapeutic surgery in those with occult metastases. Our patients with negative cytology without resection showed poor survival, though the lack of timely chemotherapy was likely a contributing factor. We found that atypical cytology was associated with poor outcomes, though its clinical significance remains unclear. Further research that allows for either patient matching based on treatment methods or a comparison of diagnostic strategies paired with a standardized treatment plan is needed to better understand the significance of atypical cytology and to assess which patients would most benefit from invasive staging.
